# Sub-national prevalence survey of tuberculosis in rural communities of Ethiopia

**DOI:** 10.1186/s12889-019-6620-9

**Published:** 2019-03-12

**Authors:** Daniel G. Datiko, Ermias Amsalu Guracha, Elias Michael, Girum Asnake, Meaza Demisse, Sally Theobald, Olivia Tulloch, Mohammed A. Yassin, L. E. Cuevas

**Affiliations:** 1 REACH ETHIOPIA, Box 303, Hawassa, Ethiopia; 2 Addis Continental School of Public Health, Addis Ababa, Ethiopia; 3 Liverpool School of Tropical Medicine, Pembroke Place L5 3QA, Liverpool, UK; 4 Overseas Development Institute, London, UK; 5 The Global Fund, Geneva, Switzerland

**Keywords:** Smear positive pulmonary TB cases, Prevalence, Ethiopia

## Abstract

**Background:**

Tuberculosis is a major public health problem with varying prevalence in different settings. National prevalence surveys provide evidence for planning and decision making. However, they lack the capacity to estimate subnational magnitude that affected the capacity to make selected intervention based on the prevalence. Ethiopia is among high TB burden countries with estimated prevalence of 108 per 100,000 population varying by regions. We aimed to study sub national prevalence of smear-positive TB in rural communities of southern Ethiopia.

**Methods:**

This cross-sectional study, enrolled community members aged over 14 years who had cough of at least two weeks duration. Two sputum samples were collected and examined by using smear microscopy.

**Results:**

38,304 eligible people were enumerated (10,779 from Hadiya, 10,059 from Gurage and 17,466 from Sidama) and indentified 960 presumptive cases. 16, 14 and 14 smear-positive pulmonary TB cases were identified respectively. The point prevalence of smear-positive TB were 148 per 100,000 population (95% CI: 91–241) in Hadiya, 139 per 100,000 population (95% CI: 83–234) in Gurage and 80/100,000 population (95%CI: 48–135) in Sidama zone. Gurage zone had the highest prevalent to notified cases of seven to one.

**Conclusions:**

The prevalence of smear positive TB varies by districts and is high in rural southern Ethiopia compared to the estimated national prevalence. More TB patients remain missed and unreached, impacting negatively on health outcomes. TB case finding approaches should be revisited and innovative approaches and tools to identify missing people with TB should be scaled up.

## Background

Tuberculosis (TB) is one of the major public health problems claiming millions of lives. It is the first leading infectious cause of death [1–4].However, a third of estimated cases, close to four million people with TB are missed globally contributing to continued disease transmission and mortality [5–7]. The Global Plan to End TB underscores the importance of identifying missing people with TB and reaching 90% of estimated TB case in the general population and among key populations [8, 9].

National prevalence surveys are very important to inform better estimation of TB burden in a country and to demonstrate the success or failure of TB prevention and control efforts over the last decades. Repeated national surveys would provide important information on the progress and performance of national programs. The reduction in TB prevalence reported from some countries could be the result of improved access to diagnostic and treatment services. The increased access facilitated by community-based interventions was synergistic to active case finding activities in the community, which increased the detection of symptomatic individuals who otherwise may not have accessed the services. [10, 11].

Ethiopia has been implementing the DOTS strategy since 1995. TB services have been decentralized to the community. However, Ethiopia, missed about 60,000 people with TB in 2016, contributes to about 3 % of missed people with TB globally and remains among 30 high burden countries, 3rd in Africa [12].TB continues to be the leading cause of morbidity and mortality in the country among in-patients and children, accounting for 4.3% of all-causes of mortality [11].

Prevalence surveys conducted at sub-national levels in Ethiopia reported variation by regions within the range of 90–256 per 100,000 population with higher rates of undiagnosed or untreated people with TB in the pastoralist and rural communities [11, 13–17]. According to the first National TB Prevalence survey in 2011, the prevalence of bacteriologically confirmed TB was 108 /100,000 population. The population of southern region and pastoralist communities was reported to have higher prevalence than other population in Ethiopia [18]. However, this was not accompanied by interventions to improve the TB programme performance in such settings. In addition, there was no study conducted at subnational or district level to see variations of TB prevalence in the country. The aim of this study is to determine the sub national prevalence of smear positive pulmonary TB in southern region of Ethiopia.

## Methods

### Study design

This is a cross sectional study conducted in the three rural districts of southern Ethiopia. The study was conducted from 2012 to 2013.

### Sample size estimation

The sample size was estimated using the single population survey formula for adult smear positive. The prevalence was estimated to be 0.17% for Hadiya and Gurage in2013 and 0.26% for Sidama in 2012 based on the WHO estimates for Ethiopia [1, 11], 95% confidence interval (CI), margin of error of 0.1%, and a design effect of 1.5. Ten percent was added to compensate for households that could not be reached during the study period. The calculated sample size was 10,005 participants for Hadiya and Gurage, and 14,880 for Sidama. Given the estimated 3.5 adults per household, the number of households to be visited was 2859 for Hadiya and Gurage and 4251 for Sidama zones.

### Study areas

**Fig. 1:**
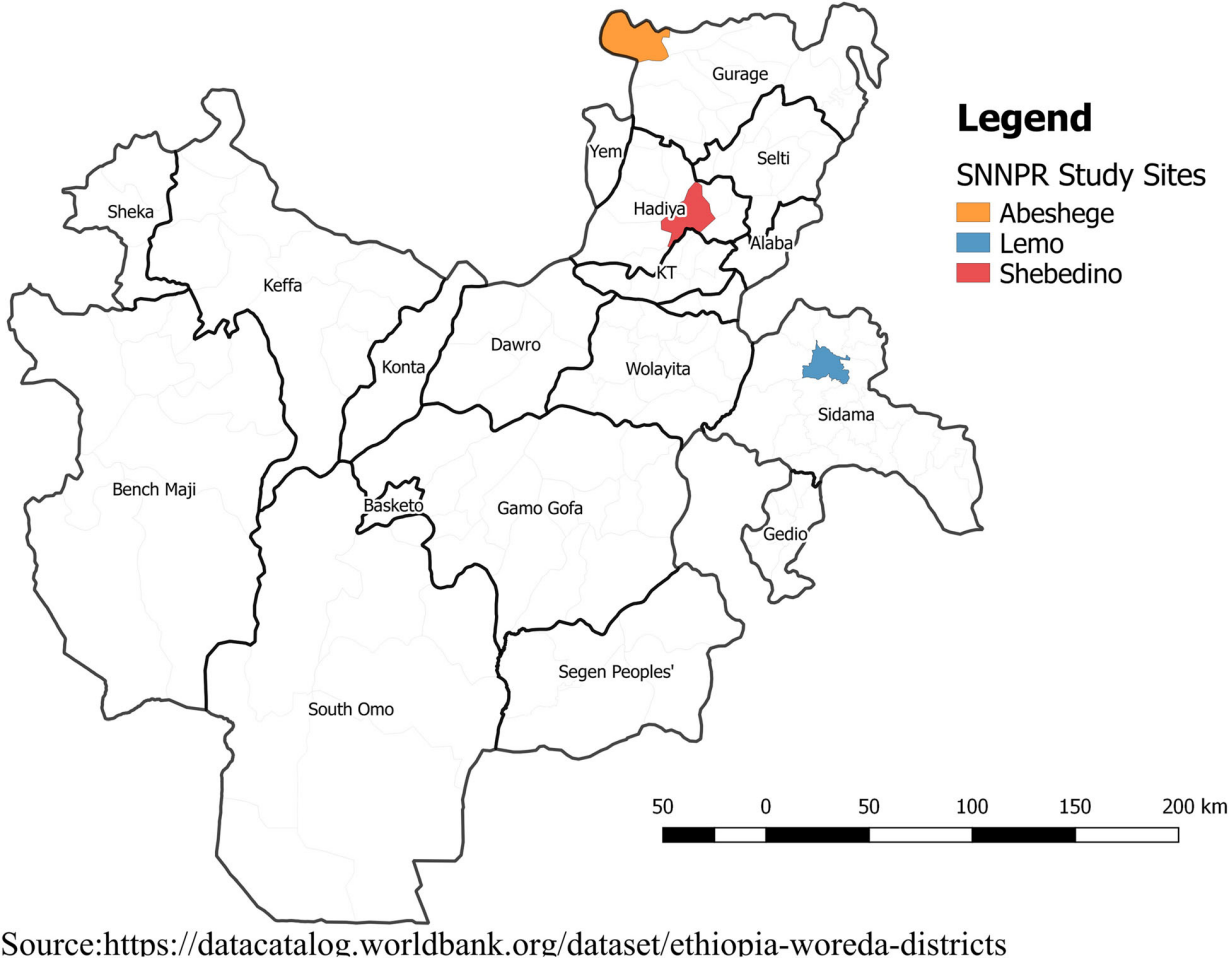
Map of the Sub National Tuberculosis Prevalence Study Areas in Southern Ethiopia

Southern region has a population of about 18 million and 93% of the population lives in rural communities. DOTS was started in 1995 and decentralized to health centres and communities. Diagnostic and treatment services are provided in hospitals and most health centres while health posts provide DOT in the community. The prevalence surveys were conducted to measure the prevalence of smear positive pulmonary TB after two years of implementing innovative community based approach in Sidama zone (Wave I TB REACH Grant) [19] and as baseline in Gurage and Hadiya zones for scale up of the intervention (Wave III TB REACH Grant). The districts (Fig. 1) and catchment village population of the study area were randomly selected for Gurage and Sidama zones. In Hadiya zone, we included all villages where previous prevalence survey had been conducted ten years earlier [17].

**Abeshige district** is located in Gurage zone. It has a population of 73,817 living in 26 kebeles (the lowest administrative unit with an average population of five thousand). The study was conducted in seven villages of the district after 19 years of DOTS implementation.

**Lemo district** is located in Hadiya zone. It has a population of 144,244 living in 33 kebeles. The study was conducted in five communities with a population of 21,224 people. A prior prevalence survey was conducted in the districts ten years before this current follow up study. A previous prevalence study was conducted in 2003 after eight years of DOTS implementation.

**Shebedino district** is located in Sidama zone. It has a population of 261,128 living in 32 kebeles. This district is among the 19 districts of Sidama zone where innovative community-based TB intervention funded by TB-REACH initiative was implemented for two years. The study was conducted in five villages with 17, 466 people. Description of the study population is shown in Tables1 and 2.

### Data collection and analysis

We trained high school completed residents and health workers from the study areas about how to conduct house-to-house visits, screen household members and organize sample collection. Pretested semi-structured questionnaires were used for data collection. The questionnaire includes sociodemographic variables (age, sex, residence, family size and economic condition), TB symptoms and risk actors (history of contact with known TB case, history of treatment, consumption of raw milk and smoking).

Households in the kebeles were enumerated. The data collectors conducted house-to-house visits and interviewed heads of the household. They registered household members and asked if anyone had cough of two weeks or more among ≥14 years old household members. Those who had productive cough of at least two weeks were instructed and requested to submit two sputum samples. Spot-morning sputum samples were collected by laboratory professionals in the community or nearby health centre. Household visit was conducted by health workers to ensure collection of two samples per presumptive case. The sputum samples were placed in cold box which was maintained at temperature of 2–8 °C and transported to the nearest health centres for sputum examination by direct microscopy using ZN method. Laboratory professionals from the three health centres conducted the sputum examination. All slides were kept for external quality assurance which was done by the regional laboratory experts at the regional laboratory. There was no discordant slide between the health centre and regional laboratory examined slides.

The supervisors conducted daily supervisory visits to the villages and checked randomly selected households to confirm they had been surveyed. The questionnaires were checked for completeness and consistency. Data entry and analyses was done using SPSS for windows version 20. Prevalence was calculated using the eligible population as a denominator. The ratio of missed to detected (prevalence to notified ratio)is calculated by using the total number of smear positive pulmonary TB cases detected by the health facilities prior to the study to the total number of smear positive cases identified by the study. The point prevalence of smear positive pulmonary TB is calculated per 100,000 population with 95% confidence interval. For the categorical variables we have done univariate regression analysis to assess the risk factor for acquiring TB. Multivariate analysis was done by fitting in the variables with *p* < 0.20. 95% CI was calculated for odds ratio (OR) adjusted for age, sex and residence. Number needed to screen is the total number of participants screened to get one smear positive case while the number needed to tests is the total number of presumptive pulmonary TB cases needed to test by microscopy to diagnose one smear positive case.

### Ethical clearance

Ethical approval was obtained from the Addis Continental Institute of Public health, Hawassa University College of Medicine and Public Health of University of Hawassa and the Ethical Review Committee of Southern Regional Health Bureau. A support letter was provided to support the conducting of field work within communities. The study participants were informed about the survey and provided verbal informed consent to participate in the study. Parents and guardians were asked and gave verbal informed consent on behalf of children under 18 years old. People with TB were treated according to the National TB guideline. Smear negative presumptive TB cases were advised to seek medical advice if their symptoms persisted.

## Results

A total of 14 kebeles (five from Hadiya, five from Gurage and four from Sidama zones) were included in the study (Table 1). The total study population was 38, 304 people. The mean age was 37 (SD + 12) for Shebedino, 32 (SD + 14) for Lemo and 35 (SD + 13) for Abeshge). The proportion of women was 45% (35.4% for Shebedino, 49.3% for Lemo and 49.5% for Abeshge) as shown in Table 3. Majority (49.1%) of the participants were farmers (31.3% for Shebedino, 40.4% for Lemo and 75.6% for Abeshge) as shown in Table 2.

**Table 1:** Description of the study area and survey coordination in three zones of the southern region of Ethiopia, 2012–2013

Characteristics	Hadiya zone, Lemo district	Gurage zone, Abeshege district	Sidama zone, Shebedino district
District population	144,244	73,817	261,128
Population density per sq. km	342.64	217.13	451.83
N^o^ of kebeles	33	28	32
N^o^ of Health centres	7	4	6
N^o^ of Health posts	33	28	32
Health service coverage	100%	95%	85%
Baseline case detection rate	43%	31%	80%
Training days for data collection	2	2	2
Data collection period	Feb 2013	Dec 2013	Feb-March 2012
Participants in data collection	40	20	34
HEWs involved for sample collection	10	14	8
Supervisors	6	5	4
Laboratory technicians	2	2	2

**Table 2:** Sociodemographic characteristics of presumptive TB cases from the three zones of the southern region of Ethiopia, 2012–2013

Characteristics		Hadiya zone, Lemo district	Gurage zone, Abeshege district	Sidama zone, Shebedino district
Number		289	357	314
Age	Mean (SD)	32(14)	35(13)	37(12)
Age group	15–24 yrs	87(30.1%)	75(21%)	39(12.4%)
	25–34 yrs	61(21.1%)	44(12.3%)	88(28%)
	35–44 yrs	57(19.7%)	78(21.8%)	56(17.8%)
	45–54 yrs	36(12.5%)	76(21.3%)	66(21%)
	55 + yrs	48(16.6%)	84(23.5%)	65(20.7%)
Gender	Male	153 (52.9%)	191(53.5%)	114(36.2%)
	Female	136 (47.1%)	166(46.5%)	201(63.8%)
Occupation	Farmer	118 (40.8%)	270(75.6%)	98(31.3%)
	Student	79 (27.3%)	45(12.6%)	86(27.5%)
	House wife	66 (22.8%)	29(8.1%)	119(37.3%)
	Others	26 (9.1%)	13(2.7%)	12(3.9%)
Marital status	Single	95(32.9%)	75(21%)	105(33.5%)
	Married	187(64.7%)	239(66.5%)	186(59%)
	Divorce	2(0.7%)	10(2.8%)	3(1%)
	Widowed	5(1.7%)	33(9.2%)	20(6.5%)
Educational status	No schooling	142 (49.1%)	120(33.6%)	199(63%)
	Schooling	147 (50.9%)	237(66.4%)	116(37.7%)

### Hadiya zone prevalence survey

Of 10,779 adults surveyed, 289(2.7%) presumptive TB cases were identified and 16 (5.5%) smear positive pulmonary TB cases were detected. Of 289 presumptive cases, 13 (4.5%) were men and 3 (0.5%) were women. 12 (92.3%) of the 13 cases were identified among contacts of other smear positive pulmonary TB patients. The number needed to screen (NNS) to identify a smear positive pulmonary TB case was 675 adults while the number needed to test (NNT) to identify a smear positive pulmonary TB case was 18 adult presumptive TB cases. The point prevalence of smear positive pulmonary TB was 148 per 100,000 population (95% CI: 91–241), 237/100,000in men [139–406] and 56/100,000 in women [95% CI: 19–166]. Nine cases with smear positive cases had been diagnosed before the survey and had started treatment. Further samples collected during the survey were reported as being smear negative by smear microscopy. For every smear positive pulmonary TB case detected, there was one undetected person with TB in the community. The ratio of prevalence to notified cases is one to one. The risk of becoming smear positive pulmonary TB is higher for men [AOR: 6.34, 95%CI (1.36–29.58)] and among contacts [AOR: 30.5, 95% CI (6.9–134.3)].

### Gurage zone prevalence survey

Of 10,059 adults surveyed, 357 (3.54%) presumptive TB cases were identified and found 14 (3.9%) people with smear positive pulmonary TB. The NNS and NNT were 719 and 45 adults respectively. The point prevalence of smear positive pulmonary TB was 139 (95% CI: 83–234) per 100,000 population. The prevalence among men was 157 (95%CI: 80–310) and 121 (95% CI: 55–263) for women. Two out of the 14 people with smear positive pulmonary TB were on treatment during the survey. For every person with smear positive pulmonary TB detected, there were seven undetected people with TB in the community. The risk of becoming smear positive pulmonary TB was higher among contacts [AOR: 4.71, 95%CI (1.3–17.1)].

### Sidama zone prevalence survey

Of 17,466 adults surveyed, 314 presumptive TB cases were identified and found 14 (4.5%) smear positive pulmonary TB cases; all of the presumptive TB cases were identified by innovative community based approach and were on the list for revisit. The NNS and NNT were 798 and 45 adults respectively. The point prevalence of smear positive pulmonary TB was 80/100,000 population (95%CI: 46–135), 67/100,000 for men (95%CI: 31–147) and 129/100,000 for women (95%CI: 66–255). Five people with smear positive pulmonary TB were on treatment during the survey. For every smear positive pulmonary TB case detected, there were two undetected in the community. The risk of becoming smear positive pulmonary TB was higher among contacts (OR: 95%CI: 30.5: 6.9–134.3). The detailed description of the background characteristics is shown in Table 3.

**Table 3:** baseline characterisation and prevalence of smear positive tuberculosis in three zones of the southern region of Ethiopia, 2012–2013

Characteristics	Hadiya zone, Lemo district	Gurage zone, Abeshege district	Sidama zone, Shebedino district
Number of villages surveyed	5	7	4
Population in the study area	21,224	73,817	33,505
N^o^ of households	4333	2050	6836
14 years above population	10,799	10,059	17,466
Men	5475 (50.7%)	5084(50.5%)	8907(50.9%)
Women	5324 (49.3%)	4975(49.5%)	6180(49.1%)
Presumptive cases	289 (2.7%)	357 (3.6%)	314 (1.8%)
Men	153(2.8%)	191 (3.8%)	114(1.3%)
Women	135(2.5%)	166(3.3%)	200(3.2%)
PTB+ cases	16	14	14
Men	13(81%)	8(57%)	6(43%)
Women	3(19%)	6(43%)	8(57%)
Point prevalence	148 [91–241]	139 [83–234]	80[48–135]
Men	237 [139–406]	157 [80–310]	67[31–147]
Women	56[19–166]	121 [55–263]	129[66–255]

## Discussion

We report higher prevalence of smear positive TB in southern region compared to the National prevalence survey. This could be due to the inadequate decentralization of DOTS services and lack of awareness in the community and inability of the TB programme to reach these missed cases. In Hadiya, the prevalence has increased from 78/100,000 (ref [20]) in 2003 to 148/100,000 in the current survey, indicating inadequate decentralization, low case notification and inability to reach missed people with TB hence contributing to sustained TB transmission.

The prevalence of TB is high in sub Saharan African and Asian countries although there is varying prevalence within communities. It is mainly fuelled by HIV epidemic in Africa and predisposing factors like smoking; and poor socioeconomic condition and poor health infrastructure in both regions which limit access to diagnosis and care. The prevalence we report is lower than reports from Pakistan 256/100,000 [13] and 638/100,000 from Zambia [20]. It is higher compared to the national prevalence survey report (The Ethiopian National TB Prevalence survey (108/100,000) and reports from south west part of the county (10.9%) [21] and (30/100,000) [22] but lower than some reports from the northern and central parts of the county (Addis Ababa (189/100,000) and Dabat district, Northwest Ethiopia (174/100,000) [23]. This could be due to difference in study period, the methodologies used, population (subnational verses national), socioeconomic condition and performance of TB control program in these settings.

Similar to other study reports, the risk of becoming smear positive case was higher among people with history of contact [17, 24, 25]. The number of unreached or missed people with TB in the community was higher among communities in Gurage zone which could be explained by inadequate decentralization of the service and limited community based TB care. However, this was lower in Sidama zone where an innovative community based approach [16] has been implemented contributing to declining TB burden meaning this zone, which is closer to the national prevalence estimate. Therefore, targeted innovative community based approaches, that reduce the barriers to seeking care are crucial to identify and treat unreached people with TB as early as possible.

Within the districts we observed difference in the proportion of women presumptive cases and number of missed TB cases. Sidama zone had higher proportion of female presumptive TB cases. This could be due to better awareness, addressed access related barriers and reduced cost and opportunity costs through the diagnosis through the community approach by making it easier to report symptoms within the confidential environment of the household and a reduction of perceived risk of gender related stigma [26]. The NNS and NNT were lowest in Hadiya which could be due to increasing prevalence, delays in seeking care and limited access. However, this requires further study.

This study indicates that one-size fits all intervention would be problematic as the magnitude of the disease varies between communities; intensive interventions may be required in areas where there is higher proportion of missed people with TB. This study demonstrates how community based interventions could play a vital role in clearing the backlog of people with TB and reaching those previously missed; although lack of baseline survey data limits the conclusion. Furthermore, the Shebedino survey could serve as a proxy indicator of the impact of active case finding in the community, implemented for two years prior to the study, in reducing the prevalence [27, 28].

Compared to the national prevalence survey, our finding showed higher prevalence as suggested by the national prevalence survey, though the diagnostic tools used were different. However, the increasing result we found in Hadiya zone suggests that there may be increase in prevalence. This study could serve as evidence indicating this trend and could be used as indirect indicator to repeating prevalence survey in Ethiopia. Sub-national surveys conducted among a similar population or geographic area may provide information on performance of the programs and changes in TB burden like the two surveys in Hadiya - conducted 10 years apart.

The prevalence in the study area could have been an underestimate because smear microscopy may have missed cases with low bacilli load. In addition, introduction of community-based approaches before the surveys may have reduced the backlog of long standing cases and the survey detected the most recent incident cases. This effect could have been interpreted as decline in the prevalence in Sidama zone. In this setting, presumptive TB cases were continuously identified and screened through a house-to-house visit for two years prior to the survey by innovative community based approach by TB REACH project [19]. The low prevalence reported from this district could be a proxy to declining in prevalence or lower TB incidence due to the intervention and long term implementation of DOTS [19, 29].

### Limitations of the study

The first limitation is the lack of baseline prevalence study makes it difficult to measure the impact of the community-based interventions. The second is the inability to compare among the studies due to difference in study period, duration and type of interventions implemented in the districts.

## Conclusion

The prevalence of smear positive pulmonary TB varies by districts and the risk factors remain similar. Community-based interventions should be promoted to improve case finding and reach missing people with TB in the region and throughout the country.

## Abbreviations

AOR: Adjusted odds ratio
CI: Confidence interval
DOTS: Directly observed treatment short course
NNS: Number needed to test
NNT: Number needed to screen
TB: Tuberculosis
WHO: World health organization
